# Process Evaluations of Interventions for the Prevention of Type 2 Diabetes in Women With Gestational Diabetes Mellitus: Systematic Review

**DOI:** 10.2196/51718

**Published:** 2025-02-06

**Authors:** Iklil Iman Mohd Sa'id, Natasha Hotung, Madeleine Benton, Iliatha Papachristou Nadal, Anisah Baharom, Matthew Prina, Barakatun Nisak Mohd Yusof, Kimberley Goldsmith, Samantha Birts, Ching Siew Mooi, Angus Forbes, Khalida Ismail, Boon How Chew

**Affiliations:** 1 Department of Family Medicine Faculty of Medicine and Health Sciences Universiti Putra Malaysia Selangor Malaysia; 2 Department of Psychological Medicine King's College London London United Kingdom; 3 Newcastle University Newcastle upon Tyne United Kingdom; 4 Department of Psychological Medicine King's College London Leeds United Kingdom

**Keywords:** gestational diabetes mellitus, randomized controlled trial, process evaluation, implementation, complex interventions

## Abstract

**Background:**

Gestational diabetes mellitus (GDM) is characterized by hyperglycemia in pregnancy and typically resolves after birth. Women with GDM have an increased risk of developing type 2 diabetes mellitus (T2DM) later in life compared to those with normoglycemic pregnancy. While diabetes prevention interventions (DPIs) have been developed to delay or prevent the onset of T2DM, few studies have provided process evaluation (PE) data to assess the mechanisms of impact, quality of implementation, or contextual factors that may influence the effectiveness of the intervention.

**Objective:**

This study aims to identify and evaluate PE data and how these link to outcomes of randomized controlled trials (RCTs) of T2DM prevention interventions for women with GDM.

**Methods:**

A systematic review was conducted to identify studies published from 2005 to 2020 aiming to capture the most recent DPIs. Five electronic bibliographic databases (Cochrane Library, Cochrane Collaboration Registry of Controlled Trials, Embase, PubMed, and MEDLINE) were searched to identify relevant studies. Inclusion criteria were published (peer-reviewed) RCTs of DPIs in women with a current diagnosis or history of GDM. Exclusion criteria were studies not published in English; studies where the target population was women who had a family history of T2D or women who were menopausal or postmenopausal; and gray literature, including abstracts in conference proceedings. The Medical Research Council’s PE framework of complex interventions was used to identify key PE components. The Mixed Method Appraisal Tool was used to assess the quality of included studies.

**Results:**

A total of 24 studies were included; however, only 5 studies explicitly reported a PE theoretical framework. The studies involved 3 methods of intervention delivery, including in person (n=7), digital (n=7), and hybrid (n=9). Two of the studies conducted pilot RCTs assessing the feasibility and acceptability of their interventions, including recruitment, participation, retention, program implementation, adherence, and satisfaction, and 1 study assessed the efficacy of a questionnaire to promote food and vegetable intake. While most studies linked PE data with study outcomes, it was unclear which of the reported PE components were specifically linked to the positive outcomes.

**Conclusions:**

While the Medical Research Council’s framework is a valuable source for conducting systematic reviews on PEs, it has been criticized for lacking practical advice on how to conduct them. The lack of information on PE frameworks in our review also made it difficult to categorize individual PE components against the framework. We need clearer guidance and robust frameworks for conducting PEs for the development and reporting of DPIs for women with GDM.

**Trial Registration:**

PROSPERO International Prospective Register of Systematic Reviews CRD42020208212; https://www.crd.york.ac.uk/PROSPERO/display_record.php?RecordID=208212

**International Registered Report Identifier (IRRID):**

RR2-https://doi.org/10.1177/16094069211034010

## Introduction

Gestational diabetes mellitus (GDM) is characterized by hyperglycemia first recognized in pregnancy and currently impacts between 8% and 23% of pregnancies globally [1]. GDM typically resolves after birth but can have significant implications for both short- and long-term health of women and babies [2]. Women with prior GDM have a nearly 10-fold increased risk of developing type 2 diabetes mellitus (T2DM) than those with normoglycemic pregnancy [3]. Risk factors for T2DM after GDM include high BMI, increasing age, multiparity, poor glucose tolerance, and prepregnancy complications [4-6]. Lifestyle interventions that target both diet and exercise are associated with small but significant effects in reducing the risk of T2DM in women with GDM [7-10].

Process evaluation (PE) theoretical frameworks provide a systematic approach to planning the design of health behavior change interventions [11,12]. However, many of the randomized controlled trials (RCTs) reported not using these PE frameworks to plan the design of lifestyle interventions [11,13-17]. Several key PE theoretical frameworks have been developed and widely used [13-17], and researchers have made progress in updating the methodologies and definitions of key PE components to construct more comprehensive frameworks to measure the success and effectiveness of interventions [13]. Knowledge of these processes can better inform future policy and practice [18] and provide opportunities to improve study design and methodologies of future diabetes prevention interventions (DPIs) [19].

The UK Medical Research Council (MRC) theoretical framework on PEs for complex interventions was chosen for this systematic review [15], as it is well regarded and recognizes the need for more formal guidance on how to conduct PEs. The framework considers processes along 3 interlinked dimensions: implementation, mechanisms of impact, and contextual factors. Table 1 presents a glossary of key PE components and methodologies [15].

The overarching aim of this review was to identify and evaluate any PE conducted and how these link to outcomes of RCTs of DPIs after GDM. Specific objectives were to identify the extent to which PE components were reported and described in RCTs of DPIs for women with previous or current GDM and to assess whether these components could contribute to explaining the intervention outcomes of the DPIs.

**Table 1:** Glossary of key process evaluation components and methodologies.

Key process evaluation components				Definitions			Quantitative methods			Qualitative methods
**Implementation**										
	Content delivered		The content included in the intervention and the method in which the content is delivered to the participants.			Structured observation			Observational study	
	Method of content delivery		How the intervention content was delivered to the participants and the extent to which quantitative and qualitative methods were used.			Structured observation			Observational study	
	Fidelity		The extent to which the intervention was delivered as intended. Fidelity represents the quality and integrity of the intervention.			Behavioral coding systems Questionnaires Protocol checklist Structured observation			Audiotapes of sessions Observational study	
	Dosage delivered		The quantity or number of intended units delivered during the intervention including the component delivered and the extent of the participant’s engagement.			Checklist records of dose delivered Structured observation			Audiotapes of sessions	
	Dosage received		The quantity or number of intended units received during the intervention including the component received by the participants.			Behavioral coding systems Questionnaires Structured observation Digital monitoring (eg, digital feedback)			Audiotapes of sessions Focus groups Interviews Observational monitoring	
	Adaptations		The extent to which alterations were made to an intervention to achieve better contextual fit.			Structured observation			Observational study	
	Reach		The proportion of the intended target audience that comes into contact with and participated in the intervention.			Attendance lists Standardized protocols			Observational monitoring	
**Mechanisms of impact**										
	Participant responses toward the intervention	The responses of participants who interacted with and received the intervention, their satisfaction, and the degree to which they found the intervention acceptable.			Questionnaires			Interviews Focus groups		
	Mediators	The extent to which intermediary processes inform subsequent changes in outcomes.			Structured observations			Observational study		
	Unexpected pathways or consequences	The extent to which identifying unexpected pathways and mechanisms during the intervention meets the research needs and leads to intervention outcomes.			Structured observations			Observational study		
**Contextual factors**										
	Barriers	Any external factors that may act as a barrier toward the intervention implementation or its effect on the outcomes.			Statistical analysis Questionnaires			Interviews Focus groups Observational study		
	Facilitators	Any external factors that may act as a facilitator to the intervention implementation or its effect on the outcomes.			Statistical analysis Questionnaires			Interviews Focus groups Observational study		

## Methods

### Study Design

This review used the PRISMA (Preferred Reporting Items for Systematic Reviews and Meta-Analyses) guidelines and was registered on PROSPERO (CRD42020208212). Owing to limited guidance on conducting systematic reviews in this area, we used the UK MRC theoretical framework from which we derived a glossary of components to use as a checklist to extract and categorize qualitative and quantitative data (Table 1) [15]. This framework considers 3 linked dimensions in which to consider processes: implementation, mechanisms of impact, and contextual factors. Implementation involves an assessment of fidelity, dose delivered and received (ie, how often or effectively it was delivered), adaptations made, reach, content delivered, and method of content delivery [20,21]. Mechanisms of impact involve an assessment of participants’ satisfaction with the intervention, “unexpected consequences,” and “mediators” [18]. Contextual factors refer to the external barriers and facilitators, such as cultural or organizational factors, that may alter the implementation of an intervention [22].

### Search Strategy

The timeframe for the search was limited to between December 1, 2005, and December 16, 2020. This timeframe was selected because of the rapid increase in DPIs in addition to the fast-moving space of digital technology. The review aimed to capture the most recent DPIs. Five electronic bibliographic databases were used: Cochrane Library; Cochrane Collaboration Registry of Controlled Trials; Embase; PubMed; and MEDLINE. The main key search terms were GDM, RCT, and PE (see Multimedia Appendix 1 for a detailed list of search terms and strings and Multimedia Appendix 2 for key definitions). Reference lists of the included studies were also searched for additional eligible studies. Boolean search was used to combine the keywords with operators such as AND, NOT, and OR to further produce more relevant results, for example: ((GDM OR gestational diabetes OR pregnancy-induced diabetes OR diabetes in pregnancy) AND ((RCT OR controlled clinical trial OR pragmatic control trial OR clinical trial) AND (process evaluation OR program evaluation OR process assessment OR process acceptance OR outcome measures)); ((GDMs OR gestational diabetes OR pregnancy-induced diabetes)) AND ((RCT OR controlled clinical trial OR pragmatic clinical trial OR clinical trial) OR (process evaluation OR program assessment OR process acceptance OR outcome measures).

### Eligibility Criteria

Inclusion criteria were published (peer-reviewed) RCTs of DPIs in women with a current diagnosis or history of GDM. Exclusion criteria were studies not published in English; studies where the target population was women who had a family history of T2D or women who were menopausal or postmenopausal; and gray literature, including abstracts in conference proceedings. For further details about the eligibility criteria, please see the study protocol [23].

### Study Selection

After initial deduplication of the extracted data using Endnote (Clarivate), studies were uploaded to the platform Rayyan for screening. The 2 reviewers (IIMS and MB) independently screened the titles and abstracts of the studies. Disagreements were resolved via consensus or decided by a third reviewer (IPN) when consensus could not be reached. The full texts of potentially relevant studies were retrieved for further screening and independently appraised by 2 reviewers (IIMS and MB) for final inclusion.

### Data Extraction, Study Characteristics, and Analysis

Standardized data extraction forms were developed and piloted. Extracted data included a summary of study characteristics, evaluation of processes (if any), methods, and findings. Data were extracted by a single reviewer (IIMS) and verified by a second reviewer (NH); discrepancies were again resolved via consensus.

We conducted a narrative synthesis of the studies as we anticipated that the heterogeneity of the methods used to assess processes would preclude meta-analysis, as has been found previously [10].

### Assessment of Quality

The Mixed Method Appraisal Tool was used to assess the quality of included studies by 2 independent reviewers (IIMS) and (NH). The discrepancies were again resolved via consensus.

## Results

### Study Selection

**Figure 1:**
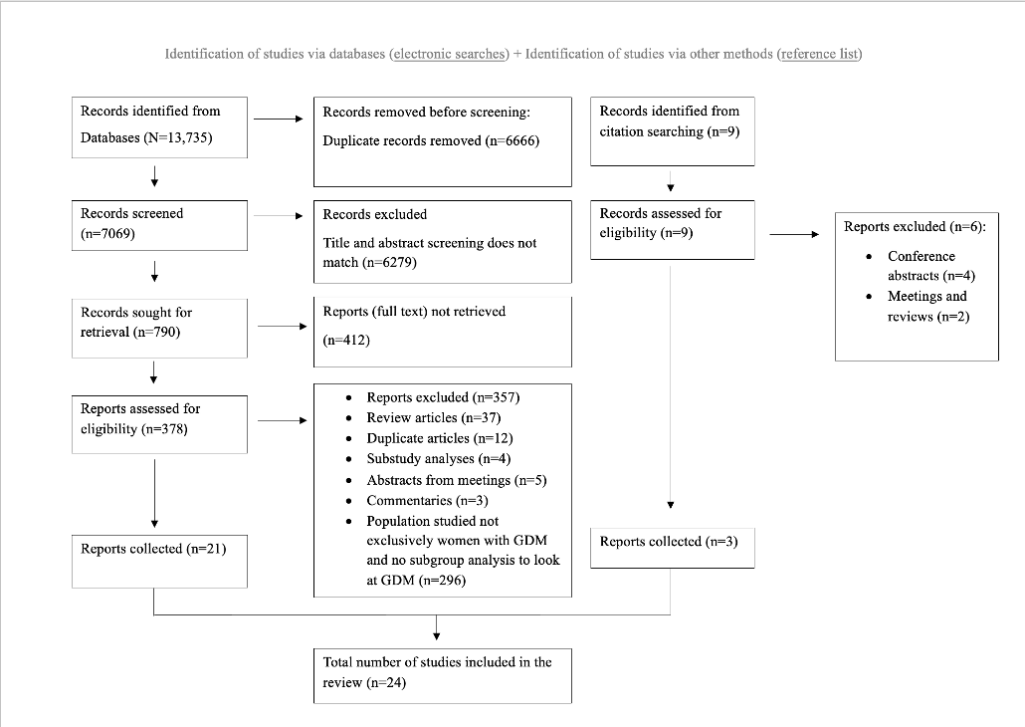
PRISMA (Preferred Reporting Items for Systematic Reviews and Meta-Analyses) diagram of search. GDM: gestational diabetes mellitus.

The PRISMA flow diagram for this review is shown in Figure 1. Of the 13,735 records initially identified, the full text of 378 full-text studies were screened for eligibility and 21 studies met the eligibility criteria. An additional 3 studies were added following citation searching. Therefore, 24 studies were included in this review. Most of these studies fulfilled at least 3 methodological quality criteria outlined by the Mixed Method Appraisal Tool for each study design (Multimedia Appendix 3 [3,24-46]).

### Study Characteristics

The stated primary aims of all 24 DPI studies reviewed were to improve dietary and physical activity outcomes, increase self-efficacy levels and risk perception of T2DM, and decrease maternal postpartum weight and BMI. The 24 studies evaluated in-person (n=8), digital (n=7), and hybrid (n=9) interventions. Two of the studies were pilot studies of the feasibility and acceptability of DPIs. All studies were published between 2007 and 2020. Most studies (n=22, 92%) were conducted in high-income countries. Only 2 studies were conducted in low- to middle-income countries: Egypt (n=1) and Malaysia (n=1). The sample size ranged from 31 to 1180 participants. The intervention duration varied from 12 weeks to 6 years. See Multimedia Appendix 4 [3,24-46] for a description of study characteristics of the studies included in this review.

### Number and Type of PE Components Reported

The most frequent PE components evaluated were content delivered and method of content delivery (n=24), dose delivered (n=20), reach (n=20), dose received (n=17), barriers (n=15), facilitators (n=8), and participants’ responses toward the intervention (n=8). Relatively few studies reported process data relating to adaptations (n=7), mediators (n=7), fidelity (n=5), or unexpected pathways or consequences (n=2). All PE components outlined by the MRC framework and reported in the reviewed DPIs are summarized in Multimedia Appendices 5 and 6 [3,24-46].

### PE Frameworks and Measures Reported

Five studies explicitly referred to a PE framework or PE quantitative or qualitative measures. Only 1 of these referred to process measures in the context of the MRC framework of complex interventions [24]. The others reported measures to ensure adherence to the study protocol [25], to measure “reach” (using the penetration, implementation, participation, and effectiveness metric) [26,27], or to assess participants’ satisfaction to the program using a survey [47].

### Implementation

#### Content Delivered and Method of Content Delivery

Eight studies evaluated in-person or group DPIs [3,28-34], 7 evaluated digital DPIs [35-40,48], and 9 delivered hybrid DPIs [24-27,41-44]. Most of the interventions were multicomponent that focused on diet and self-directed physical activity [24-27,33,35,40-46,49] or were facilitator led [29,30,43]. One intervention also included advice on breastfeeding [28].

#### Behavior Techniques and Theoretical Frameworks Used to Deliver Content

Behavior change techniques included motivational interviewing (MI) with individual goal setting [26,29]; enhancing healthy lifestyle change and self-management [24]; guided self-help [33]; nutritional and physical activity recommendations [30]; self-help guidance based on responses to a dietary food intake questionnaire [3]; and guideline-based nutritional strategies [44]. Five interventions were underpinned by established theoretical frameworks—social cognitive theory and the transtheoretical model of behavior change [25]; the Health Action Process Approach supported by social cognitive and self-regulation theory [27] based on a Health Belief Model [50]; and messages on weight loss, diet, and physical activity based on a harm reduction model to promote healthy lifestyle changes [43].

The link between a process and outcome was reported in 2 studies. In the first, the use of behavior change strategies was reported to improve women’s knowledge, beliefs, and self-reported practices as well as decrease postpartum weight gain in 1 study [33]. In the second study, there were no observed changes in weight, waist circumference, or blood glucose level and this was correlated with no changes in health perception or self-efficacy [3].

#### Fidelity

Five DPIs (digital: n=3 and hybrid: n=2) reported on the fidelity (quality) of intervention implementation [25,27,36,39,45]. Assessment of fidelity involved recording telephone calls or sessions [36,45], a qualitative [45] or quantitative [36,39] assessment of adherence, and sometimes also involved input from the participants [45]. Two of the studies attributed a high level of fidelity to effective weight loss and weight goals [27,36].

#### Dose Delivered

Nineteen studies reported on the quantity, frequency, and duration of the interventions [24-26,28-30,32,34,36,39-43,48]. Most studies delivered around 1 to 4 sessions lasting 2 hours. The shortest dose duration was 12 weeks and included 12-weekly sessions of 2.5 hours including group physical activity, health education, and an individualized lifestyle counseling session [29].

Further details in relation to dose delivery were generally limited. For instance, the frequency [24,40] or quantity of sessions or emails [43,45] were not reported. Similarly, 3 digital DPIs of web-based interventions involving health websites [40], internet telemedicine [37], or pedometer messaging [38] did not report the quantity of health content and messages sent by health care professionals. Borgen et al [35] reported the delivery of their mobile health app but did not mention how long it would take to peruse the app’s content. Finally, another web-based intervention [44] did not report on how frequently participants should use the pedometer or step count goal target.

#### Dose Received

Sixteen of the studies reported data on the dose received by the participants [3,24-26,28-30,32,36-38,43,45]. The dose response included the average number of sessions attended by women [25-27,29,36,40,43-45]; those returning documents or questionnaires [51-53]; combined food intake and physical activity logbooks [38,54,55]; and log-ins to websites or systems [37,40,45].

Information on the dose received was used to highlight the willingness of participants to take part in the study and the subsequent success of the intervention. For example, in 1 hybrid study [44], it was reported that most women in the study attended all 4 lifestyle counseling sessions in their intervention, concluding that it was successful in reducing weight and increasing physical activity levels. Details on the dose received were also used to explain any lack of intervention effects. In another hybrid study [43], authors attributed their unsuccessful intervention outcomes to the limited number of women logging onto their website intervention and observed that engagement did not improve with fortnightly automated email reminders. One digital study observed that they did not collect data on user engagement for its mobile health app to protect women’s privacy and questioned that this may have explained the nonsignificant effect of the intervention [35].

#### Adaptations

Adaptations were described in 7 DPIs, including 2 in-person [38,55], 2 digital [35,45], and 3 hybrid DPIs [24,25,32]. These adaptations were seen as important components for the success of the intervention and included providing a choice of intervention methods [28] and tailoring interventions to the characteristics of the target population [45] to specific ethnic or cultural backgrounds [24,34,35]. In 3 hybrid DPIs, MI techniques were used to tailor sessions for participants [24,25] and illustrations and simple messages were added to address low health literacy [41].

#### Reach

Twenty studies reported on the reach of its intervention in relation to the target population [24-26,28-32,34,36-41,43,45]. Loss to follow-up was a recognized challenge, perhaps reflecting the case mix of the target population. Two of the studies referred to pregnancy complications as a reason for dropout [31,33] with another attributing loss to follow-up to work or personal commitments, initiation of a weight loss diet, subsequent pregnancy, not being contactable, and finding intervention resources unhelpful [40]. One digital DPI reported a low participation rate of 17%, making inference to the broader target population difficult [39]; another reported that few women who received information about their intervention proceeded to enroll for participation [38]. Other studies that used PE methods to measure reach reported low rates [24,27,43] and 2 additional studies had 33% to 39% attrition rates [29,34].

### Mechanisms of Impact

#### Participant Responses Toward the Intervention

Eight studies reported on participant feedback and responses toward the interventions including 4 digital [35,38-40], 3 hybrid [25,26,44], and 1 in-person DPI [29]. Overall, women expressed satisfaction with the interventions [25,29,38,39,44], reporting increased confidence in setting health goals [39,40] and more engagement with health management [35].

Some studies did not receive a positive response. Only a third of women reported being motivated by the website content in 1 digital intervention [40] and even fewer (22% and 31%) had a positive reaction to the text messaging component. Women also criticized the lack of information on optimal carbohydrates to consume when transitioning between pregnancy to postpartum diets and reported the need for more low-fat recipes [25,40].

#### Mediators

Mediators were described in the interventions in 2 in-person DPI studies [29,34], 4 digital DPIs [35,37,39,40], and 1 hybrid DPI [43], although not explicitly. O’Dea et al [29] found that women valued individual face-to-face sessions with health care professionals when setting health goals which improved their stress levels, diet self-efficacy, and quality of life. Social relationships also developed between the women due to regular attendance at the same lifestyle counseling groups [34]. Digital DPIs were also reported to increase women’s engagement in their own health [35], enhance self-efficacy and confidence [37,40], and provide reassurance in developing and attaining health goals [39]. Further, 1 hybrid study [43] qualitatively linked the inclusion of partners in their intervention as a key mediator for women making them more likely to participate in and engage with the intervention.

#### Unexpected Pathways or Consequences

Only 2 studies [39,41] reported unexpected consequences in relation to their interventions. Carolan-Olah and Sayakhot [41] observed there was an unusually high percentage of women in the intervention group who had attended their postpartum oral glucose tolerance test (OGTT) appointment. Although not directly linked to the study, the intervention highlighted the importance of postpartum testing, which may have increased women’s motivation to attend future follow-up appointments. Another digital study [39] experienced unexpected challenges with data retrieval from women and general practitioners, resulting in missing baseline blood tests and self-reported data at follow-up. However, this missing data did not appear to negatively influence the findings as women in the intervention group were found to significantly reduce their fat intake.

### Contextual Factors

#### Barriers

Fifteen studies referred to barriers that women faced when participating in interventions [3,24-26,29,31,33-35,37-40,43,44]. Such barriers included lack of time due to family and work commitments, subsequent pregnancy, and changes to daily life due to the demands of motherhood [24,29,31,33,40,44] technology issues, and lack of internet access [24,38,43]. Similarly, unavailability due to childcare responsibilities and forgetting to attend study visits prevented mothers from engaging in in-person lifestyle counseling sessions in the nondigital interventions [3,24,29]. Another in-person DPI acknowledged how culture plays a role in hindering women from participating in and completing an intervention when they are placed under pressure to commit to family and social norms [34].

#### Facilitators

The 8 DPI studies including 4 in-person DPIs [28,29,31,34], 2 hybrid DPIs [43,44], and 2 digital DPIs [37,40] described facilitators as contextual factors which may have influenced the outcomes of their interventions. The most common facilitator identified in 1 hybrid and 1 in-person DPI was receiving support from a partner during the intervention [29,43] and having higher levels of income and personal education background [43]. Having access to healthy food, food vouchers, a babysitter, and an exercise buddy were also reported as facilitators to assist and support the uptake and maintenance of postpartum healthy behaviors [31,40]. The competency and skills of health care professionals delivering the interventions were acknowledged as facilitators to ensure the uptake of the intervention [28]. Having an accessible, central location for assessments and employers who allowed time for visits also facilitated participation [34,44].

## Discussion

### Overview

We identified and reviewed 24 RCTS of DPIs that evaluated at least 1 PE component for women with previous or current GDM. Only 10 studies explicitly reported individual processes, and only 5 explicitly referred to a PE framework or PE quantitative or qualitative measures to assess processes. Overall, few studies reported and evaluated processes in relation to study outcomes, and although most DPIs linked PE components and DPI outcomes in some capacity, it was challenging to attribute any 1 process component to the effectiveness and success of the intervention.

### Summary of Key Findings

The most complete of the PE components reported across all 24 studies was the combined component of content delivered and method of content delivery. Clear narratives were provided on how the interventions were delivered and their content. Most of the interventions also referred to broad behavior change techniques such as goal setting and MI, which are known to be effective in promoting lifestyle behavior change [56,57]. However, few studies linked these theories with mechanisms of action by providing a detailed psychological or behavior change theoretical framework on which their intervention was underpinned. Such frameworks are important to make sense of “how,” “why,” and “under what circumstances” intervention components work together to achieve the desired outcomes. Moving forward, we recommend that all RCTs of DPIs publish an overview of the theories they have used to design and evaluate the intervention for greater transparency.

Although most DPIs reported information on the dose delivered*,* PE measures on the dose received were less complete. Generally, increased frequency and longer sessions resulted in more engagement with the intervention and better outcomes [26-28,30,31,36]. More frequent sessions may also be preferred by the participants: for a study on the use of MI and hemoglobin A_1c_ outcomes, women said that they would like more sessions to improve their engagement with the intervention [58]. The timing of the intervention is also a consideration. Most of the DPIs that we reviewed delivered interventions during either the antenatal or postpartum periods. However, systematic review evidence suggests that optimal pregnancy outcomes can be achieved by delivering the intervention during both periods since engagement with a healthy lifestyle poses tends to be poorer during the postpartum stage [5,51]. Moreover, there was little information in the studies reviewed on how the dose received impacted the success of the intervention. The challenges in assessing adherence to the dose received are well recognized as people may have received a dose but choose not to take it [59]. This is also supported in the literature where a nurse-led psychological intervention for a T2D cluster RCT study identified a lower dose received than intended, but that this was not associated with the dose delivered [60]. Further research to explore how timing, frequency, and duration of the intervention affect outcomes among women with current or previous GDM is indicated.

Although all 24 studies included in this review sought to describe the reach of the intervention, few gave a detailed evaluation of this measure. Poor reach was attributed to generic reasons, most commonly low participation rates, loss to follow-up, and small sample sizes [26,27,29,36,39]. The increase in OGTT appointment attendance found in 1 study [41] is contrary to prior research linking loss to follow-up and withdrawal numbers to participants’ reluctance to attend OGTT appointments [49,54], which highlights the influence that different contexts have on outcomes in this population. Moreover, the studies that made adaptations to tailor their DPIs to personal or cultural needs [24,25,28,34,35,41,45] are likely to have increased reach by making the interventions more relevant. A clear definition of the target population and consideration of how the DPI can be adapted to make it as inclusive as possible seems important for its success.

Most of the studies reported on barriers to intervention engagement. Similar to previous systematic reviews [10,61], the barriers primarily related to lack of time, childcare commitments, and challenges to maintaining healthy lifestyles [24,29]. Focus groups with women suggest that these inherent barriers can be mitigated by combining direct contact with health care professionals with web-based interventions [48]. Moreover, it is worth noting that, despite the many barriers to participation identified, women were still in favor of the DPIs, and many resulted in positive outcomes. This aligns with previous research in T2D [62] and supports a need and willingness among women with previous or current GDM to engage with DPIs.

A particular concern identified by this review was the lack of reporting or evaluation of fidelity. Only 5 studies reported on this process component. Ensuring fidelity is a key aspect of any DPI because it safeguards against nonadherence to the study protocols and inadequate implementation delivery [47,63]. Ideally, fidelity should be measured prior to conducting full-scale implementation to distinguish between outcomes that are related to ineffectiveness from those related to protocol deviation [64,65]. In our review, methods to assess fidelity included audiotaping and patient registers of attendance [27,36]. Such methods have been tried and tested in the T2D setting [47,63] and are likely to be more robust than relying on the expertise of health care professionals as a fidelity measure [66]. To minimize research waste, fidelity assessments should be commenced at the design stage of a trial and incorporate standardized measures where possible.

Finally, we welcome the use of both quantitative and qualitative methods to evaluate the PE components in our studies because this enables a more in-depth understanding of the relationship between individual components and outcomes [52,67]. We reemphasize the importance of mixed methods when conducting thorough PE.

### Strengths and Limitations

To the best of our knowledge, this is the first and only systematic review of PEs of T2DM prevention interventions for women with GDM. We used robust methodology, an established framework of PE for complex interventions [15], and restricted the review to RCTs, which are known to be the best design for evaluating complex health care interventions [15]. The glossary list of standardized DPI content and PE terms developed during the review process can be used to guide future RCTs in this area. However, while the MRC framework [18] is a valuable source for conducting systematic reviews on PEs, it has been criticized for lacking practical advice on how to conduct them [68]. The lack of information on PE frameworks in our review also made it difficult to categorize individual PE components against the framework. Moreover, there is a lack of consensus on how PE components should be defined and interpreted by researchers, which is only partially addressed in the MRC guidelines [15], which facilitate understanding of PE constructs but provide little clarity on what should be measured and how.

### Conclusions

This systematic review has highlighted that there are important gaps in the reporting of PE metrics for RCTs of T2DM in women with GDM. We recommend rigorous, systematic, and in-depth PE guidance to facilitate reporting of these studies. Future research should focus on reaching consensus on the reporting of PE measures using established frameworks and evaluating PE in real-world health care settings to optimize the interpretation of study outcomes.

## Appendix

Multimedia Appendix 1 Detailed list of search terms and strings.

Multimedia Appendix 2 Key definitions of types and formats of DPI content delivered, behavior change techniques, and psychological theoretical frameworks embedded within DPIs. DPI: diabetes prevention intervention.

Multimedia Appendix 3 Quality assessment of review articles.

Multimedia Appendix 4 Study characteristics of interventions for the prevention of type 2 diabetes in women with gestational diabetes mellitus.

Multimedia Appendix 5 Process evaluation implementation components for T2DM prevention lifestyle intervention studies on women with GDM. GDM: gestational diabetes mellitus; T2DM: type 2 diabetes mellitus.

Multimedia Appendix 6

Multimedia Appendix 7 PRISMA Checklist for systematic review.

## Abbreviations

DPI: diabetes prevention intervention
GDM: gestational diabetes mellitus
MI: motivational interviewing
MRC: Medical Research Council
OGTT: oral glucose tolerance test
PE: process evaluation
PRISMA: Preferred Reporting Items for Systematic Reviews and Meta-Analyses
RCT: randomized controlled trial
T2DM: type 2 diabetes mellitus

## Data Availability

All data generated or analyzed during this study are included in this published article (and its supplementary information files).
