# Supplementary material for: Process Evaluations of Interventions for the Prevention of Type 2 Diabetes in Women With Gestational Diabetes Mellitus: Systematic Review
Source: Interact J Med Res. 2025 Feb 6;14:e51718. doi: 10.2196/51718 (PMC11843062; doi:10.2196/51718)
Supplement: Multimedia Appendix 1 [file ijmr_v14i1e51718_app1.docx]

**KEYWORDS: GDM AND (RCT or process evaluation)**

1. **Gestational diabetes mellitus**
2. Gestational diabetes
3. Diabetes in pregnancy
4. Gdm
5. Pregnancy diabetes mellitus
6. Pregnancy induce diabetes
7. Diabetes mellitus gravidarum
8. **Randomized Controlled Trial**
9. Randomized Controlled Trial
10. Randomised Controlled Trial
11. Controlled Clinical Trial
12. Pragmatic Clinical Trial
13. Pragmatic study
14. Pragmatic studies
15. Equivalence Trial
16. Clinical Trial
17. Randomization
18. Randomisation
19. Random Allocation
20. Allocated
21. Double-Blind Method
22. Double Blind Procedure
23. Double-Blind Studies
24. Single-Blind Method
25. Single Blind Procedure
26. Single-Blind Studies
27. Control Groups
28. Control Group
29. (random* or sham or placebo*)
30. ((singl or doubl*) adj (blind* or dumm* or mask*))
31. ((tripl* or trebl* or adj (blind* or dumm* or mask*))
32. (Nonrandom* or non random* or quasi-random* or quasirandom*)
33. ((open label or open-label) adj5 (study or studies or trial*))
34. ((equivalence or superiority or non-inferiority or noninferiority) adj3 (study or studies or trial*))
35. ((pragmatic or practical) adj3 trial*)
36. ((quasiexperimental quasi-experimental) adj3 (study or studies or trial*))
37. **Process evaluation**
38. Program evaluation
39. Evaluation mechanism
40. Process assessment
41. Process acceptance
42. Process measure
43. Outcome measure
44. Risk reduction intervention

**KEYWORDS ENTRY LINE BY LINE**

1. Gestational diabetes mellitus
2. Gestational diabetes
3. Diabetes in pregnancy
4. Gdm
5. Pregnancy diabetes mellitus
6. Pregnancy induce diabetes
7. Diabetes mellitus gravidarum
8. 1 OR 2 OR 3 OR 4 OR 5 OR 6 OR 7
9. Randomized Controlled Trial
10. Randomised Controlled Trial
11. Controlled Clinical Trial
12. Pragmatic Clinical Trial
13. Pragmatic study
14. Pragmatic studies
15. Equivalence Trial
16. Clinical Trial
17. Randomization
18. Randomisation
19. Random Allocation
20. Allocated
21. Double-Blind Method
22. Double Blind Procedure
23. Double-Blind Studies
24. Single-Blind Method
25. Single Blind Procedure
26. Single-Blind Studies
27. Control Groups
28. Control Group
29. (random* or sham or placebo*)
30. ((singl or doubl*) adj (blind* or dumm* or mask*))
31. ((tripl* or trebl* adj (blind* or dumm* or mask*))
32. (Nonrandom* or non random* or quasi-random* or quasirandom*)
33. ((open label or open-label) adj5 (study or studies or trial*))
34. ((equivalence or superiority or non-inferiority or noninferiority) adj3 (study or studies or trial*))
35. ((pragmatic or practical) adj3 trial*)
36. ((quasiexperimental quasi-experimental) adj3 (study or studies or trial*))
37. 9 OR 11 OR 12 OR 13 OR 14 OR 15 OR 16 OR 17 OR 18 OR 19 OR 20 OR 21 OR 22 OR 23 OR 24 OR 25 OR 26 OR 27 OR 28 OR 29 OR 30 OR 31 OR 32 OR 33 OR 34 OR 35 OR 36
38. Process evaluation
39. Program evaluation
40. Evaluation mechanism
41. Process assessment
42. Process acceptance
43. Process measure
44. Outcome measure
45. Risk reduction intervention
46. 38 OR 39 OR 40 OR 41 OR 42 OR 43 OR 44 OR 45 (PE)
47. 8 AND 37 (GDM AND RCT)
48. 37 OR 46 (RCT OR PE)
49. 8 AND 48 (GDM AND RCT OR PE)
50. 9 OR 10 OR 11 OR 12 OR 15 OR 16 OR 17 OR 18 OR 19 (SPECIFIED RCT)
51. 8 AND 50 (GDM AND RCT)
52. 46 OR 50 (PE OR RCT)
53. 8 AND 52 (GDM AND PE OR RCT)
54. REMOVE DUPLICATES FROM 53
