# Supplementary material for: Process Evaluations of Interventions for the Prevention of Type 2 Diabetes in Women With Gestational Diabetes Mellitus: Systematic Review
Source: Interact J Med Res. 2025 Feb 6;14:e51718. doi: 10.2196/51718 (PMC11843062; doi:10.2196/51718)
Supplement: Multimedia Appendix 2 [file ijmr_v14i1e51718_app2.docx]

| **FORMAT OF CONTENT DELIVERY** | **Definitions** |
| --- | --- |
| In-person DPI | Involving the delivery of face-to-face clinic-based health education programs and lifestyle counselling/coaching sessions |
| Digital DPI | Involving the delivery and use of any e-health including the internet, mobile health app, telephone, websites, emails and use of a pedometer |
| Hybrid DPI | Involving the delivery of both in-person and digital content |
| **FORMAT OF CONTENT DELIVERY** |  |
| Interactive | HCPs involved in the delivery of the DPI via communication with the patient |
| Self-guided | Automated self-help materials either as printed, digital or hybrid |
| **TYPES OF CONTENT DELIVERED** |  |
| Health education program | Dietitian or nurse delivering information and/or education to promote healthy diet, weight loss and physical activity |
| Physical activity program | Physiotherapist or sports instructor delivering information and/or education and/or activities such as on exercise, a walking group, or using a pedometer |
| Nutritional program | Dietitian delivering information and/or education and/or coaching in healthy diet and weight loss. |
| Psychological program | Dietitian or nurse delivering lifestyle counselling or coaching including behavioural change techniques to support health education program |
| **BEHAVIOUR CHANGE TECHNIQUES** |  |
| Goal-setting | Assessment of lifestyle behaviours, examining values and motivation, setting measurable goals, defining action plans, identifying barriers and facilitators to help patients maintain sustainable lifestyle changes and remove barriers to achieve their health goals (Michie et al, 2013) [50]. |
| Implementation intentions (II) | If-then plans that spell out in advance how one wants to achieve a set goal. Implementation intentions has been found to enhance the rate of goal attainment (Gollwitzer and Oettingen, 2020) [67]. |
| Motivational interviewing (MI) | A guiding style of communication which involves following (listening) and directing (providing information or advice) to empower patients to change health behaviour (Miller and Rollnick, 2012) [70]. |
| Patient centred counselling | Psychological counselling centred around the patient who chooses topics to discuss with an HCP according to their preferences and perceived needs (Neuner-Jehle et al, 2013) [71] |
| Question behaviour effect (QBE) | An effect referring to the influence of any type of questioning including questions about intentions and predicted future behaviour (Wilding et al, 2016) [76]. |
| **THEORETICAL FRAMEWORKS** |  |
| Health action process approach (HAPA) | The adoption, initiation, and maintenance of health behaviours explicitly conceived as a process that consists of at least a motivation phase and a volition phase (Schwarzer, 2016) [74] |
| Health belief model (HBM) | Health-related behaviours predicted by a person’s motivation and can be divided into three categories: individual perceptions, modifying factors, and likelihood of action (Janz and Becker, 1984) [69] |
| Self-regulation theory (SRT) | The use of self-efficacy as a pathway to experience greater agency which leads to increased feelings of motivation, effort, persistence and learning when making lifestyle changes (Bandura, 1991) [66]. |
| Social cognitive theory (SCT) | The change of the main causal determinant of behaviour (i.e. self-efficacy) using four techniques: mastery experiences, modelling or vicarious experience, persuasion and giving physiologically compatible experiences (Schunk and Usher, 2012) [73]. |
| Transtheoretical model (TTM) | Six stages of modifying behaviour by integrating principles and theories of behaviour change, including precontemplation, contemplation, preparation, action, maintenance and termination (Grimley et al, 1994) [68] |
