# Supplementary material for: Process Evaluations of Interventions for the Prevention of Type 2 Diabetes in Women With Gestational Diabetes Mellitus: Systematic Review
Source: Interact J Med Res. 2025 Feb 6;14:e51718. doi: 10.2196/51718 (PMC11843062; doi:10.2196/51718)
Supplement: Multimedia Appendix 3 [file ijmr_v14i1e51718_app3.docx]

| **Studies** | **Criteria from the Mixed Method Appraisal Tool (MMAT)** | | | | | | | | | | | | | | | | | | | | | | | | |
| --- | --- | --- | --- | --- | --- | --- | --- | --- | --- | --- | --- | --- | --- | --- | --- | --- | --- | --- | --- | --- | --- | --- | --- | --- | --- |
|  | **1.1** | **1.2** | **1.3** | **1.4** | **1.5** | **2.1** | **2.2** | **2.3** | **2.4** | **2.5** | **3.1** | **3.2** | **3.3** | **3.4** | **3.5** | **4.1** | **4.2** | **4.3** | **4.4** | **4.5** | **5.1** | **5.2** | **5.3** | **5.4** | **5.5** |
| Borgen et al  (2019) [35] | - | - | - | - | - | 🗸 | 🗸 | - | 🗸 | 🗸 | - | - | - | - | - | - | - | - | - | - | - | - | - | - | - |
| Carolan-Olah and Syakhot (2019) [41] | - | - | - | - | - | - | 🗸 | 🗸 | - | 🗸 | - | - | - | - | - | - | - | - | - | - | - | - | - | - | - |
| Ferrara et al  (2011) [25] | - | - | - | - | - | - | - | - | - | - | - | - | - | - | - | - | - | - | - | - | 🗸 | 🗸 | 🗸 | 🗸 | 🗸 |
| Ferrara et al  (2016) [36] | - | - | - | - | - | 🗸 | 🗸 | 🗸 | - | 🗸 | - | - | - | - | - | - | - | - | - | - | - | - | - | - | - |
| Holmes et al  (2018) [24] | - | - | - | - | - | - | - | 🗸 | - | 🗸 | - | - | - | - | - | - | - | - | - | - | - | - | - | - | - |
| Homko et al  (2007) [37] | - | - | - | - | - | - | - | 🗸 | - | 🗸 | - | - | - | - | - | - | - | - | - | - | - | - | - | - | - |
| Hu et al  (2012) [42] | - | - | - | - | - | - | 🗸 | 🗸 | 🗸 | - | - | - | - | - | - | - | - | - | - | - | - | - | - | - | - |
| Kim et al  (2012) [38] | - | - | - | - | - | 🗸 | - | - | - | 🗸 | - | - | - | - | - | - | - | - | - | - | - | - | - | - | - |
| Koivusalo et al  (2015) [28] | - | - | - | - | - | 🗸 | - | 🗸 | 🗸 | 🗸 | - | - | - | - | - | - | - | - | - | - | - | - | - | - | - |
| Lipscombe et al  (2019) [26] | - | - | - | - | - | 🗸 | 🗸 | 🗸 | - | 🗸 | - | - | - | - | - | - | - | - | - | - | - | - | - | - | - |
| Liu et al  (2018) [46] | - | - | - | - | - | - | 🗸 | 🗸 | - | 🗸 | - | - | - | - | - | - | - | - | - | - | - | - | - | - | - |
| McManus et al (2018) [43] | - | - | - | - | - | - | - | 🗸 | - | 🗸 | - | - | - | - | - | - | - | - | - | - | - | - | - | - | - |
| Nicklas et al  (2014) [45] | - | - | - | - | - | - | 🗸 | 🗸 | - | 🗸 | - | - | - | - | - | - | - | - | - | - | - | - | - | - | - |
| O'Dea et al (2015) [29] | - | - | - | - | - | - | - | - | - | - | - | - | - | - | - | - | - | - | - | - | 🗸 | 🗸 | 🗸 | - | 🗸 |
| O’Reilly et al  (2016) [27] | - | - | - | - | - | - | - | - | - | - | - | - | - | - | - | - | - | - | - | - | - | 🗸 | 🗸 | - | 🗸 |
| Peacock et al  (2014) [44] | - | - | - | - | - | 🗸 | - | 🗸 | 🗸 | 🗸 | - | - | - | - | - | - | - | - | - | - | - | - | - | - | - |
| Pérez-Ferre et al (2015) [30] | - | - | - | - | - | - | 🗸 | 🗸 | - | 🗸 | - | - | - | - | - | - | - | - | - | - | - | - | - | - | - |
| Reinhardt et al (2012) [39] | - | - | - | - | - | - | 🗸 | 🗸 | - | 🗸 | - | - | - | - | - | - | - | - | - | - | - | - | - | - | - |
| Rollo et al  (2020) [40] | - | - | - | - | - | - | - | - | - | - | - | - | - | - | - | - | - | - | - | - | 🗸 | 🗸 | 🗸 | - | 🗸 |
| Shek et al  (2014) [31] | - | - | - | - | - | - | 🗸 | 🗸 | - | 🗸 | - | - | - | - | - | - | - | - | - | - | - | - | - | - | - |
| Shyam et al (2013) [32] | - | - | - | - | - | 🗸 | - | 🗸 | 🗸 | 🗸 | - | - | - | - | - | - | - | - | - | - | - | - | - | - | - |
| Tawfik (2016) [33] | - | - | - | - | - | - | - | 🗸 | - | 🗸 | - | - | - | - | - | - | - | - | - | - | - | - | - | - | - |
| Vézina-Im (2019) [3] | - | - | - | - | - | - | 🗸 | - | - | 🗸 | - | - | - | - | - | - | - | - | - | - | - | - | - | - | - |
| Zilberman-Kravits et al (2019) [34] | - | - | - | - | - | - | - | 🗸 | - | 🗸 | - | - | - | - | - | - | - | - | - | - | - | - | - | - | - |
