# Supplementary material for: Process Evaluations of Interventions for the Prevention of Type 2 Diabetes in Women With Gestational Diabetes Mellitus: Systematic Review
Source: Interact J Med Res. 2025 Feb 6;14:e51718. doi: 10.2196/51718 (PMC11843062; doi:10.2196/51718)
Supplement: Multimedia Appendix 4 [file ijmr_v14i1e51718_app4.docx]

**Table S1: Study characteristics of interventions for the prevention of type 2 diabetes in women with gestational diabetes mellitus**

| **No** | **Authors (year), country** | **Intervention**  **type** | **Age (mean; yrs)** | **Sample**  **size** | **Intervention vs control group** | **Intervention duration** | **Follow up time points** | **Primary & secondary outcome measures** | **Study Results** | **PE Components Reported** | **Methods used to conduct PE** |
| --- | --- | --- | --- | --- | --- | --- | --- | --- | --- | --- | --- |
| 1 | Borgen et al (2019) [35], Norway | Digital | 54% aged  30-27 | Iv: 115  C: 123 | Iv: Pregnant+ mobile health app  C: Standard care: Info on healthy diet & PA; guidance on when to measure BG levels. | 7 mths | 0, 7 mths | *Primary outcome(s)*  2-hr blood glucose level of routine postpartum OGTT | No difference found for 2-hr blood glucose level postpartum OGTT; 6.7 mmol/L (95% CI 6.2 to 7.1) in Iv group and 6.0 mmol/L (95% CI 5.6 to 6.3) in C group | *content delivered*  *method of content delivery; adaptations; mediators; barriers* | Self-reported questionnaires to measure perceived health while using an app |
| 2 | Carolan-Olah and Sayakhot (2019) [41], Australia | Hybrid | 31.5 | Iv: 52  C: 58 | Iv: Online educational programme modules; 15-30 mins duration to peruse content  C: Standard care: 90-min health education session | 12 wks | 0, 12 wks | *Primary outcome(s)*  Maternal weight loss  *Secondary outcome(s)*  Maternal BMI; BP; glycaemic level; infant weight at birth | Statistically significant differences reported for Iv vs C group in maternal weight and glycaemia post-Iv. More women in Iv group reported weight loss post-Iv (90.4% vs 48.3%, p<0.0001). | *content delivered;*  *method of content delivery; dose delivered; adaptations; reach;* *unexpected pathways/*  *consequences* | Not reported |
| 3 | Ferrara et al (2011) [25], United States | Hybrid | Not reported | Iv: 96  C: 101 | Iv: Telephone & in-person counselling sessions  *Prenatal phase:*  1 in-person session*;* 2 telephone calls  *Early postpartum phase:* 2 in-person sessions; 7-10 telephone sessions  *Late postpartum phase:* 3 telephone counselling calls  C: Printed educational materials & newsletter on infant safety; standard care. | 12 mths | 0, 6 wks, 7 & 12 mths post-partum | *Primary outcome(s)*  Postpartum weight loss  *Secondary outcome(s)*  Dietary fat intake; PA; breastfeeding | Proportion of women reaching postpartum weight goal in Iv group (vs C group) higher, but not statistically significant (37.5 vs 21.4%, absolute difference 16.1%; *p* = 0.07) | *content delivered;*  *method of content delivery; dose delivered; dose received; adaptations; reach; participant responses; barriers* | Self-monitoring diaries (fat gram intake and mins of PA); satisfaction with Iv questionnaire. |
| 4 | Ferrara et al (2016) [36],  United States | Digital | 38% aged 30-34 | Iv: 1087  C: 1193 | Iv: Printed materials on healthy BMI, 30 mins of daily PA and healthy eating; tailored letter on goals for gestational weight gain; 13 session guidebook to review via telephone with lifestyle coach and dietitian  C: Printed educational materials with lifestyle recommendations; standard care. | 12 mths | 0, 6 wks, 6 & 12 mths | *Primary outcome(s)*  Clinic-measured postpartum weight  *Secondary outcome(s)*  Vigorous-intensity activity; energy intake; hypertension; depression | Iv group had, on average, significantly higher odds of meeting weight goals vs  women in C group (odds ratio [OR] 1.28; 95% CI 1.10–1.47). | *content delivered;*  *method of content delivery; fidelity; dose delivered; dose received; reach* | Audiotaped telephone sessions; 10% of telephone sessions coded using a checklist of Iv components to assess fidelity to protocol; food frequency and pregnancy PA questionnaires |
| 5 | Holmes et al (2018) [24],  Northern Ireland  *PE framework referenced* | Hybrid | 33.7 | Iv: 29  C: 31 | Iv: 60-min educational session programme; pedometer (at least 3000 steps and 30 mins of walking per day; 12-week membership in SW; supportive texts and phone calls.  C: 60 min health education session & DVD on how to manage GDM; standard care. | 18 mths | 0, 6 mths | *Primary outcome(s)*  Weight loss 6-mths after randomisation  *Secondary outcome(s)*  Fasting glucose; 2-hr OGTT glucose; waist circumference; BMI; pedometer counts | Iv group had significant weight loss and reduction in BMI at 6 mths compared with the C group (mean ±SD, 3.9 ± 7.0 kg vs 0.7 ± 3.8 kg; *p* = 0.02). | *content delivered;*  *method of content delivery; dose delivered; dose received; adaptations;*  *reach; barriers* | Focus group sessions; pedometer “steps per day” log; food intake & PA questionnaires; 7-day food diary |
| 6 | Homko et al (2007) [37], United States | Digital | 29.5 | Iv: 32  C: 25 | Iv: Web screens including HCP info, medications and education provided; link to educational GDM materials.  C: Monitor BG levels & record insulin doses; perform foetal movement counting 3x/day; standard care. | 20 mths | 0, every 2 wks until 36 wks’ gestat-ion, weekly there-after | *Primary outcome(s)*  Self-efficacy  *Secondary outcome(s)*  Birthweight; LGA; GA at delivery; Apgar score | No significant difference between Iv and C group in fasting, postprandial blood glucose values, or pregnancy outcomes. Women in Iv group experienced increased feelings of diabetes, psychosocial efficacy. | *content delivered;*  *method of content delivery;*  *dose delivered;*  *dose received;*  *reach; mediators;*  *barriers; facilitators* | Telemedicine data entry page; paper logbooks to record self-reported blood glucose levels, insulin doses and episodes of hypoglycaemia. |
| 7 | Hu et al (2012) [42],  China | Hybrid | 32.3 | Iv: 586  C: 594 | Iv: 6 face to face meetings with dietitians in 1^st^ year;  2 additional sessions with dietitians and 2 telephone calls in 2^nd^ year.  C: Oral & written info on diabetes awareness & lifestyle modification; standard care | 24 mths | 0, 12 & 24 mths | *Primary outcome(s)*  Development of diabetes  *Secondary outcome(s)*  Changes in BMI, body fat, waist circumference, plasma glucose, insulin, HbA1C | During 1^st^ year, average body weight loss was 1.40 kg (2.1%) in Iv group vs 0.21 kg (0.3%) in C group (*P* = 0.001). The decrease was more significant among baseline overweight women in the Iv (2.91 kg/4.2%) vs C group (0.51 kg/0.7%) (*p* < 0.001) | *content delivered;*  *method of content delivery; dose delivered* | Questionnaire on major lifestyle changes (dietary & PA); 3 day 24-hr food records. |
| 8 | Kim et al (2012) [38],  United States | Digital | 31 | Iv: 21  C: 28 | Iv: Web-based education; pedometer messaging; internet forum.  C: Not given any additional materials/info; standard care | 13 wks | 0, 13 wks | *Primary outcome(s)*  Change in fasting plasma glucose and 2-hr glucose levels on a 75-g OGTT between baseline & 13-wk follow up  *Secondary outcome(s)*  Weight & behavioural constructs (self-efficacy, social support, risk perception) | At 13-wk follow up, Iv group did not have significant changes in behavioural constructs, PA or anthropometrics compared to women in the C group. Changes in fasting plasma glucose (-0.046 mmol/l vs. 0.031 mmol/l, *p* = 0.65), 2-h glucose values (-0.48 mmol/l vs. -0.42 mmol/l, p = 0.91) and weight (=0.14 kg vs. -1.5 kg, *p* =0.13) | *content delivered;*  *method of content delivery; dose delivered; dose received; reach; participant responses; barriers* | Pedometer data graphs showing individualised step count; women’s forums and online message boards. |
| 9 | Koivusalo et al (2016) [28],  Finland | In-person | 33 | Iv: 284  C: 289 | Iv: One 2-hr group counselling session led by dietitian; 3 individualised counselling sessions  C: General info leaflets on diet and PA by local antenatal clinics; standard care | 6 yrs | Baseline visit: 13.3wks gestation  2^nd^ visit: 23.1wks gestation  3^rd^ visit: 35.1wks gestation | *Primary outcome(s)*  Incidence of GDM (1+ pathological glucose value in a 75-g, 2-hr OGTT) | Reduction in incidence of GDM by 39% in high-risk group of pregnant women. | *content delivered;*  *method of content delivery; dose delivered; dose received; adaptations;*  *reach; facilitators* | Food frequency questionnaire; self-reported time spent weekly on moderate to intense PA; dietary index based on food frequency questionnaire |
| 10 | Lipscombe et al (2019) [26], Canada | Hybrid | 36.7 | Iv: 106  C: 25 | Iv: One in-person education visit with ADAPT-M coach; 12 scheduled telephone sessions on educational topics and coaching.  C: Standard care; educational handout regarding post-GDM guidelines; standard care. | 24 wks | 0, 12 * 24wks | *Primary outcome(s)*  Recruitment; participation; retention; programme implementation metrics, programme adherence; satisfaction | A low-intensity short-term telephone-based diabetes prevention programme customised for postpartum women can be feasibly delivered within standard care and is associated with comparable retention and adherence rates in postpartum women with recent GDM | *content delivered;*  *method of content delivery; dose delivered;*  *dose received;*  *reach; participant responses; barriers* | Food frequency & PA questionnaires; diet and PA records; coach logs; participant charts; programme satisfaction surveys |
| 11 | Liu et al  (2018) [46],  China | Hybrid | 32 | Iv: 586  C: 594 | Iv: 6 face to face sessions with study dietitians and 2 telephone calls in 1^st^ year; 2 individual sessions and 2 telephone calls in each subsequent year  C: General oral and written diabetes info, dietary modification and PA; standard care. | 12 mths | 0, 12 mths | *Primary outcome(s)*  Change in body weight or %reduction in initial weight from baseline to 1^st^ year  *Secondary outcome(s)*  Changes in waist circumference, body fat, energy intake, and PA from baseline to 1^st^ year | Weight loss in women with a history of GDM; mean weight loss 0.82 kg (1.12% of initial weight) in Iv group vs 0.09 kg (0.03% of initial weight) in the C group (*p* = 0.001) | *content delivered;*  *method of content delivery; dose delivered; dose received; reach* | Changes in lifestyle questionnaire (diet & PA); 3-day 24-hr food record |
| 12 | McManus et al (2018) [43], Canada | Hybrid | 34.1 | Iv: 89  C: 81 | Iv: One-on-one FDD-branded healthy living seminar; access to a password-protected, FDD-branded website; invitation to a 1-hour weekly walking group  C: Hard copy of the contemporary postpartum healthy living handout; standard care | 12 mths | 0, 3 & 12mths | *Primary outcome(s)*  7% weight reduction at 1-yr postpartum.  *Secondary outcome(s)*  A1C, demographics, programme participation & retention, programme engagement (predictors & rates) | Iv group no more likely to lose 7% of their postpartum weight at 12mths than C group. Weight, BMI and waist measures did not differ | *content delivered;*  *method of content delivery;*  *dose delivered;*  *dose received;*  *reach; mediators; barriers;*  *facilitators* | Food frequency questionnaire to document food choices |
| 13 | Nicklas et al  (2014) [45],  United States | Digital | 33.2 | Iv: 36  C: 39 | Iv: 12 core modules tailored for women with GDM; telephone & email sessions with lifestyle coach & dietitian trained in counselling; pedometers; membership to gym for 10 months; body weighing scales; measuring cups and spoons; quantity not reported  C: Handout during recruitment; no additional info to support weight loss; standard care. | 12 mths | 0, 6 mths, 12mths | *Primary outcome(s)*  Change in body weight at 12 mths from 1^st^ postpartum measured weight; self-reported pre-pregnancy weight  *Secondary outcome(s)*  Calorie intake; PA | Women in the Iv group lost a mean of 2.8 kgs from 6 wks to 12 mths postpartum and were closer to their pre-pregnancy weight at 12 mths postpartum | *content delivered;*  *method of content delivery;*  *fidelity; dose delivered; dose received; adaptations; reach* | Recorded telephone sessions; online forms; verbal reporting; anonymous website traffic data. |
| 14 | O’Dea et al (2015) [29],  Ireland | In-person | 30.5 | Iv: 24  C: 26 | Iv: Initial individualised assessment; 12 weekly sessions of 2.5 hours/week including a 1-hour group exercise programme, group education seminar and one-to-one MI session  C: Educational pamphlets for reducing diabetes risks; routine follow-up by general practitioner; standard care | 12 wks | 0, 12 wks, 12mths | *Primary outcome(s)*  Mean change in FPG levels from baseline to 1-yr follow up  *Secondary outcome(s)*  Mean change in 2-hr post load glucose tolerance, insulin resistance, diet adherence, weight, waist circumference, PA, fitness, lipid profile, & measures of mood, cognition and well-being | At 1-yr follow-up, Iv group showed significant improvements in stress, diet self-efficacy and QoL. No evidence of intervention effect on measures of biochemistry or anthropometry; the effect on one health behaviour, diet adherence, was close to significance. | *content delivered;*  *method of content delivery; dose delivered; dose received; reach; participant responses; mediators; barriers;*  *facilitators* | Digitally recorded semi-structured interviews |
| 15 | O’Reilly et al (2016) [27],  Australia | Hybrid | 34 | Iv: 284  C: 289 | Iv: One individual session; 5 group sessions during intensive phase; 2 additional follow-up telephone calls for each woman during maintenance phase  C: Standard care | 12 mths | 0, 6, 9 & 12 mths | *Primary outcome(s)*  Change in fasting blood glucose, waist circumference and weight at 12-mths | Iv superior to standard care in preventing postnatal weight gain in women with GDM; **-**1kg weight difference - potential to be significant for reducing diabetes risk | *content delivered;*  *method of content delivery; dose delivered; dose received; fidelity;*  *reach* | Audio-recorded telephone sessions |
| 16 | Peacock et al (2015) [44],  Australia | Hybrid | 36 | Iv: 16  C: 15 | Iv: One pedometer linked to a tailored web-based programme (*Stepping Up to Health*); 4 weekly nutrition coaching workshop (4 1-hour group weekly sessions).  C: Formed a wait-list group; a nutrition workshop; standard care. | 3 mths | 0, 3 mths | *Primary outcome(s)*  Weight loss from baseline to 3 mths  *Secondary outcome(s)*  Change in baseline to 3 mths for hip and waist, diet quality, WEL overall & domain scores, mins of PA per week, glucose & HOMA-IR & body FM and FFM | Iv - Effective in supporting weight loss, waist circumference, PA, and dietary habits but no significant changes in glucose metabolism or body composition; Median (IQR) results for: weight: I −2.5 (2.3) kg versus C +0.2 (1.6) kg (*p* = 0.009), waist: I −3.6 (4.5) cm versus C −0.1 (3.6) cm (*p* = 0.07)), and hip: I −5.0 (3.3) cm versus C −0.2 (2.6) cm (*p* = 0.002). | *content delivered;*  *method of content delivery; dose delivered;*  *dose received;*  *reach; participant responses; barriers;*  *facilitators* | Questionnaires (dietary & PA); pedometer step-count logged on website account |
| 17 | Pérez-Ferre et al (2014) [30],  Spain | In-person | 35 | Iv: 126  C: 111 | Iv: 2-hr group session at first visit (7-12 weeks postpartum); group and individual sessions delivered during a period of 10 weeks between 3-6 months post-delivery; moderate intensity exercise (50-60 mins 4 days/week); 1-hr reinforcement sessions added after monitored PA period  C: Recommendations on diet & PA for at least 150 min per week; standard care | 3 yrs | 0, 3 & 6mths and 1, 2 and 3 yrs | *Primary outcome(s)*  Clinical changes & biochemical parameters | Lower proportion of women in the Iv group (42.8%) developed glucose disorders at the end of the 3-yr follow-up period compared with C group (56.8%), *p* < 0.05. | *content delivered;*  *method of content delivery;*  *dose delivered;*  *dose received;*  *reach* | Semi-quantitative questionnaire (food intake); monthly evaluation by physiotherapist (PA); logbook (PA). |
| 18 | Reinhardt et al (2012) [39],  Australia | Digital | 32.6 | Iv: 18  C: 20 | Iv: 10 individualised phone-based sessions for 5 weeks, then monthly for 5 months; session duration 10–30 mins  C: Standard care | 6 mths | 0, 6 mths | *Primary outcome(s)*  Body mass index; diet; PA | Iv group significantly reduced total fat intake, total carb intake and glycaemic load and increased leisure PA compared to the c group but no significant change in physical activity levels occurred. | *content delivered; method of content delivery; dose delivered; fidelity;*  *reach; participant responses;*  *mediators;*  *unexpected pathways/ consequences;*  *barriers* | Food and PA diaries; facilitator guidebook; questionnaire (food intake & PA levels) |
| 19 | Rollo et al  (2020) [40],  Australia | Digital | 33.5 | Iv: 13  C: 14 | Iv: 5 website content sections:  1.Managing my risk  2.My plan  3.Eating  4.PA  5.Wellbeing  6 individual telehealth coaching sessions delivered via video call (20-30 mins each)  C: Standard care | 6 mths | 0, 3 & 6 mths | *Primary outcome(s)*  Feasibility including recruitment, retention, preliminary efficacy  *Secondary*  *outcome(s)*  Acceptability | Website largely acceptable and useful for women with recent GDM; no statistically significant effect on weight change and diabetes risk reduction at group level but 16 women (53% of Iv completers) lost weight at the 6-mth time point including 4 women in the Iv arms who lost ≥5% of their baseline body weight. | *content delivered;*  *method of content delivery;*  *dose delivered;*  *dose received;*  *reach; participant responses;*  *mediators;*  *barriers; facilitators* | PE survey of acceptability; food & PA questionnaire. |
| 20 | Shek et al (2014) [31],  China | In-person | 39 | Iv: 225  C: 225 | Iv: Individual lifestyle counselling sessions  C: Standard care | 36 mths | 0, twice every 3 mths, then every 6 mths | *Primary outcome(s)*  Risk of developing T2DM | Fewer women in the Iv group developed diabetes (15% vs 19%) – not statistically significant. Lower incidence among women ≥40 yrs | *content delivered;*  *method of content delivery; reach; barriers; facilitators* | Food & PA self-reported records |
| 21 | Shyam et al  (2013) [32], Malaysia | In-person | Not reported. | Iv: 39  C: 38 | Iv: Structured one-to-one sessions; individualised dietary sheet; a GI component teaching women to substitute high GI foods with low GI foods; colour coded booklets including food refs; quantity not reported  C: One-to-one session with research nutritionist; conceptual guidance to achieve conventional recommended diet (CHDR); moderate PA for 30mins (5 times/week); 4-colour coded blue for CHDR take home booklets; standard care | 6 mths | 0, 3 and 6 mths | *Primary outcome(s)*  BMI; weight; 2HPP after administration of 25g OGTT | 6mths reductions in body weight, BMI and waist-to-hip ratio in Iv (low GI) group (*p* < 0.05); mean BMI changes were significantly different between groups (Iv vs C: -0.6 vs. 0 kg/m, *p* = 0.03); More subjects achieved weight loss ≥5% in Iv group (33% vs 8%, P = 0.01). Changes in 2-hr postprandial plasma glucose were significantly different between groups (Iv vs C: median (IQR): -0.2(2.8) vs. +0.8 (2.0) mmol/L, P=0.025). | *content delivered;*  *method of content delivery; dose delivered; dose received; reach* | Food self-reported records |
| 22 | Tawfik (2017) [33],  Egypt | In-person | 31 | Iv: 103  C: 98 | Iv: Health belief model (HBM) on knowledge, beliefs, self-practices, and gestational and postpartum weight in women with GDM  C: Standard care | 9 mths | 24wks gestation6wks post-partum | *Primary outcome(s)*  Women’s knowledge; beliefs; self-reported practices; GWG; postpartum weight retention | Iv group: Proportion with high knowledge and beliefs scores increased from <50% to >70% (*p* < 0.001); more women with excessive BMI in the Iv group (65%) compared to the control group (11.6%) were meeting recommended gestational weight gain (*p* < 0.001), and postpartum weight (37.7% vs 20.3%; *p* < 0.01) | *content delivered;*  *method of content delivery; barriers* | HBM theoretical framework; questionnaire including 7 domains for knowledge, self-reported practices and beliefs (based on HBM construct) |
| 23 | Vézina-Im  et al (2019) [3],  Canada | In-person | 33.4 | Iv: 24  C: 26 | Iv: Two visits at research centre (lasting on avg. 60-75 minutes); baseline assessment and completing food frequency questionnaire; mailing at 3 months.  C: Women in the QBE group completed an intervention questionnaire (version B) that contained only the first two sections (beliefs and sociodemographic data); standard care | 6 mths | 0, 3 & 6 mths | *Primary outcome(s)*  Efficacy of a questionnaire-based implementation intentions Iv to  promote FV intake  *Secondary outcome(s)*  Weight, waist circumference, fasting glycaemia | Both groups increased their vegetable consumption compared to baseline at 3mths (*p* = 0.002) and 6mths (*p*=0.004). No significant effects on weight, waist circumference and blood glucose levels | *content delivered;*  *method of content delivery; barriers* | Food frequency questionnaire; validated self-administered questionnaire assessing food frequency and FV intake in terms of servings per day |
| 24 | Zilberman-Kravits et al (2018) [34],  Israel | In-person | 35.7 | Iv: 103  C: 77 | Iv: Three individual 45-min counselling sessions and 4 90-min group meetings  C: Education on association between GDM and increased risk of subsequent GDM pregnancies and overt diabetes; contacted by phone to build individual follow-up schedule; standard care | 24 mths | 3-4 mths post-partum | *Primary outcome(s)*  HOMA-IR at 1 & 2 yrs | Iv group: significantly improved metabolic and morphometric indices at 1 and 2yrs post-DPI; reduced insulin, glucose and HOMA-IR levels compared with those in the C group (*p* < 0.001) | *content delivered;*  *method of content delivery; dose delivered; dose received; adaptations; mediators; reach; barriers; facilitators* | Self-report food frequency & PA questionnaires |

BMI: body mass index; BP: blood pressure; CHDR: conventional recommended diet; C: Control; GDM: gestational diabetes; FV: fruit and vegetables; GI: glycaemic index; HOMA-IR: homeostatic model assessment for insulin resistance; Iv: Intervention; min: minutes; mth: month; OGTT: oral glucose tolerance test; PA: physical activity; yr: year
