# Supplementary material for: Process Evaluations of Interventions for the Prevention of Type 2 Diabetes in Women With Gestational Diabetes Mellitus: Systematic Review
Source: Interact J Med Res. 2025 Feb 6;14:e51718. doi: 10.2196/51718 (PMC11843062; doi:10.2196/51718)
Supplement: Multimedia Appendix 5 [file ijmr_v14i1e51718_app5.docx]

| **Authors, year, country, intervention type** | **IMPLEMENTATION** | | | | | | |
| --- | --- | --- | --- | --- | --- | --- | --- |
|  | **Content delivered in intervention (iv) group versus control (c) group** | **Method of content delivery during intervention** | **Fidelity** | **Dose delivered** | **Dose received** | **Adaptations** | **Reach** |
| Borgen et al [35], 2019, Norway,  Digital | Two-year Pregnant+ app intervention for women with GDM versus standard care. | Women instructed to download app at home or in hospital; the app enabled women to print out blood glucose values to support comms with HCPs; women had opportunity to write personal goals and read advs of PA during pregnancy; a link to Norwegian Diabetes Foundation was provided which included healthy recipes and pregnancy info | Not reported | Four Pregnant+ app icons; quantity not reported:  1.Blood glucose  2.Physical activity  3.Food & beverage  4.Diabetes information | Not reported | Healthy diet info was culturally adapted in the mobile health app. | Not reported |
| Carolan-Olah and Sayakhot [41], 2019,  Australia, Hybrid | One-year online web-based educational program for women with GDM versus standard care. | Women attended standard clinic-based education class and used the web-based program; first access supervised by researcher; women given URL link and log-in password for website to access at home; Quiz provided per module for women to check understanding | Not reported | Online educational program modules: 15-30 mins duration to peruse content:  1.Healthy food choices  2.Healthy habits/healthy lifestyle  3.Emotions, family & food  4.Testing blood glucose levels | Not reported | The Iv was adapted to address low health literacy and included illustrations, photos and simple messages to facilitate learning. | 52 women (47%) received the iv; results are not generalisable to other populations due to single trial centre |
| Ferrara et al [25], 2011, United States, Hybrid | A four-year Diet, Exercise and Breastfeeding Intervention (DEBI), adapted from the Diabetes Prevention Program (DPP) for women with GDM versus standard care.  DEBI comprised of 3 intervention phases including:  1. Prenatal  2. Postpartum  3. Maintenance | Two trained dieticians delivered interventions through telephone counselling; Women encouraged to follow ADA diet and engage in moderate intensity PA; women referred to a lactation consultant to encourage breastfeeding. | Explicitly stated that researchers adhered well to and showed satisfactory compliance with the protocol | Telephone & in person counselling sessions  Prenatal phase:  1.One in-person session  2.Two telephone calls  Early postpartum phase:  1.Two in-person sessions  2.7-10 telephone sessions  Late postpartum phase:  1.Three telephone counselling calls | For early postpartum phase, 74% of women completed 8 core intervention sessions; women returned a median of 3 self-monitoring diaries, 30.9% returned 6 or more; overall, women completed an average of 9.4 postpartum sessions. | Adaptation of the DPP lifestyle Iv delivered by telephone instead of face-to-face contact. | 93% of women randomised to Iv group completed the first prenatal session and 79% completed two or more sessions. |
| Ferrara et al [36], 2016, United States, Digital | A twelve-month Diabetes Prevention Program (DPP) lifestyle intervention for women with GDM versus standard care. | Women mailed printed materials and tailored letter on goals for gestational weight gain; after delivery, women offered print/telephone-based lifestyle program (modelled on DPP); women mailed 13 session guidebook to review via telephone with lifestyle coach and dietitian. | Fidelity was high (mean proportion of intervention components present = 95%, range 60-100%. | Printed materials on healthy BMI, 30 minutes of daily PA and healthy eating; tailored letter on goals for gestational weight gain; 13 session guidebook to review via telephone with lifestyle coach and dietitian. | 18.8% women completed 1-3 sessions, 16.2% completed 4-12 sessions, and 15.3% completed all 13 sessions.  Out of 1087 women, 50.3% women completed one or more telephone sessions in the iv. | Not reported | Not reported |
| Holmes et al [24], 2018, Northern Ireland, Hybrid | 18-month PAIGE postnatal educational lifestyle intervention program in overweight women with previous GDM versus standard care. | Women received educational session delivered by a HCP trained in MI; contacted intermittently by healthcare educator via text and telephone; content delivery of PAIGE took place on 2 of every 4 weeks; program delivery used learning. techniques to promote healthy lifestyle changes and self-management in line with the MRC PE framework for complex interventions. | Not reported | 60-minute educational session program delivered at time of OGTT; pedometer (at least 3000 steps and 30 mins of walking per day; 12-week membership in SW; supportive texts and phone calls. | Not reported | Each educational session was adapted using MI techniques to suit the needs of the focus group (based on socio-economic status and ethnicity). | 20 women registered and 12 completed the course in the Iv group giving a 41% adherence rate. |
| Homko et al [37], 2007, United States, Digital | A 20-month Internet-based diabetes telemedicine system for women with GDM versus standard care. | Women asked to monitor blood glucose levels daily, perform foetal movement counting 3 times a day, and record insulin doses and hypo episodes; women asked to transmit info via diabetes health network three times per week to HCPs; women received 1hr training session on how to use a computer, access websites and set up an email address. | Not reported | Web screens including HCP info, medications and education provided; link to educational materials about GDM; quantity Not reported | Seven women (22%) in the Iv group never accessed the system; six of the seven women did maintain logbooks; 28 women sent back on avg. 21.8 sets of data. | Not reported | 34 women took part in iv; two women from the Iv group withdrew from the study; rates of appt. were 90% for women in telemedicine group. |
| Hu et al [42], 2012, China, Hybrid | Two-year Tianjin GDM Prevention Program in women with prior GDM versus standard care. | Face-to-face meetings with a dietitian in the first year and two additional sessions in the second year; dietician instructed participants on modifying diet and increasing physical activity; participants completed dietary and PA habits questionnaire and completed 3 day 24hr food records five times a day assessed by dietitian. | Not reported | Six face to face meetings with dietitians to discuss how to achieve dietary and physical activity goals in first year; two additional sessions with dietitians and two telephone calls in second year. | Not reported | Not reported | Not reported |
| Kim et al [38], 2012, United States, Digital | 13-week low-intensity web-based pedometer programme in women with a recent history of GDM versus standard care.  Pedometer programme curriculum included:  1.Perception of diabetes risk.  2.Self-efficacy for weight and physical activity.  3.Benefits of and barriers to lifestyle change.  4.Self-regulatory strategies. | Iv curriculum accessible on website and messages changed daily; women received study pedometer and instructions to upload pedometer step-count data weekly; feedback displayed graphically and via text messages; access to online forum where women could interact with one another. | Not reported | Web-based education; pedometer messaging; internet forum; quantity Not reported | Women uploaded step count an average number of 1.6 **+-** 0.64 times per week; only three women posted on forum. | Not reported | 21 women participated in the Iv group, 19 attended follow up and two were lost to follow up; relatively few women who received info about Iv proceeded to access website and few proceeded to enrol for participation. |
| Koivusalo et al [28], 2016, Finland, In-person | Six-year Finnish Gestational Diabetes Prevention Study (RADIEL) moderate lifestyle intervention in pregnant women at high risk for GDM versus standard care. | Dietary counselling sessions led by dietician focused on increasing women’s intake of fruits and vegetables, fibre, low-fat and low-sugar products; women encouraged to achieve a min. of 150 mins of moderate intensity PA per week; women and study nurses planned and updated an individual PA program during follow up. | Not reported | One two hr group health education session; three individualised psychological lifestyle counselling sessions. | 26% in the Iv group met the physical activity goal of 150 min per week in the second trimester. | Counselling sessions adapted during pregnancy tailored to each woman’s personal preferences. | 144 out of 269 women participated in iv; 7 women had miscarriage or termination of pregnancy; 4 women were lost to follow up. |
| Lipscombe et al [26], 2019, Canada, Hybrid | A six-month home-based diabetes prevention program, Avoiding Diabetes After Pregnancy Trial in Moms program (ADAPT-M) for women with recent GDM versus standard care.  An intervention integrating evidence-based tailored nutrition and PA education with behaviour change techniques. | Women randomised to 1 of 3 24-week coaching interventions:  1.)PA and diet  2.)PA only  3.)Diet only;.  Regular scheduled telephone calls with assigned coach to promote behaviour change using MI, goal-setting and action planning; women coached to meet a min of 150 mins per week of moderate aerobic activity | Not reported | One in-person education visit with ADAPT-M coach; 12 scheduled telephone sessions on educational topics and coaching. | Women completed a mean 8.1**+-**3.5 or 67**+-**30% of the 12 telephone sessions. | Not reported | Penetration of target population was 17%; successfully recruited almost half of women invited; >70% of women invited retained; 79% were adherent to coaching sessions; 50 women adhered to at least 80% of the scheduled sessions; 71% achieved the recommended PA goal by 6 months. |
| Liu et al [46], 2018, China, Hybrid | One-year Tianjin Gestational Diabetes Mellitus Prevention Program in women with GDM versus standard care. | Dietitians instructed women with GDM on how to achieve the 5 goals of the iv:  1.Reduction of body weight.  2.Total fat intake <30% of energy consumed.  3.Carbohydrate 55%-65% of energy consumed.  4.Fibre intake 20 to 30g per day  5.Moderate vigorous exercise for at least 30 mins per day, 7 days per week. | Not reported | Six face to face sessions with study dietitians and two telephone calls in first year; two individual sessions and two telephone calls in each subsequent year. | Three day 24-hour food record completed by women at each clinical visit; three day 24-hour food record and self-administered questionnaire completed at first year annual visit  Quantity of dose received Not reported | Not reported | Iv completion rates were 78.5% in the Iv group. |
| McManus et al [43], 2018, Canada, Hybrid | One-year GDM-specific healthy living program, Families Defeating Diabetes (FDD) Intervention in women with recent GDM and their partners versus standard care. | Program designed to be delivered through existing diabetes education centre network; program messages and behavioural change support were enhanced by electronic media; close family member (i.e. partner) was invited to participate alongside women in the iv. | Not reported | One-on-one FDD-branded healthy living seminar; access to a password-protected, FDD-branded website; invitation to a one-hour weekly walking group. | 30 (34%) of women in Iv group attended the weekly walking group at least once; FDD website accessed by 47 women in Iv group and 13 INT male partners at least once; interaction with website was negligible with only four questions being asked during study interval and two from same woman; 28 women (and 3 male partners) accessed the website more than 10 times. | Not reported | Only 17 of participant dropouts in Iv group were due to moving away from study location. |
| Nicklas et al [45], 2014, United States, Digital | One-year web-based lifestyle program, Balance after Baby for women with recent GDM versus standard care. | Women were provided communication with life coach, weekly shopping lists, recipes & PA education; women given forms to enter weekly goals to return to pre-pregnancy weight over study period; recommended healthy dietary choices and increasing PA to > 150 mins per week; asked to watch one module per week for the first 12 weeks with 6 other optional modules available; encouraged to track diet and PA in logbooks and to continue with breastfeeding. | Study physician reviewed all recorded calls and emails to ensure adherence to patient-centred counselling techniques. | 12 core modules tailored for women with GDM; telephone & email sessions with lifestyle coach & dietitian trained in counselling; pedometers; membership to gym for 10 months; body weighing scales; measuring cups and spoons; quantity Not reported | Women watched a median of 9/12 modules, 33% watched al 12 core modules at least once and all women watched at least one module; median number of contacts with life coach was 7 (range 0-12), only one participant had no contact, 4 (range 0-9) over second 12 weeks of program, and 2 (range 0-10) over last 6 months of program. | 16 core DPP modules were adapted for the web-based Iv by subject matter experts to 12 core modules to tailor for postpartum women with GDM. | Women accessed website on all days of the week and at all times (day and night); accessed website from home, work and mobile phones; 61% of women logged dietary intake at least once and 67% used pedometers. |
| O’Dea et al [29], 2015, Ireland, In-person | A 12-week intensive group-based lifestyle program (MyAction) in women with prediabetes following GDM versus standard care. | Programme was delivered by a MDT of nurses, dietitians and PA specialists and supported by a physician; individual goal setting with HCP. | Not reported | Initial individualised assessment; 12 weekly sessions of 2.5 hours per week including a one-hour group exercise programme, a group education seminar and a one-to-one MI session | The average number of sessions attended by women in the Iv was 9.5.  Of the 24 women randomised to the Iv group, 14 (58%) participated in and completed the Iv with attendance rates of >_6; Semi-structured interviews took place with 17 women in the Iv group, where 12 completed the interviews and 5 were non-completers.  Of the 14 completers, 13 attended the EOP assessment and 14 attended the one year follow up assessment. | Not reported | Reached sample target of 54 participants but 42% randomised to Iv group did not complete the iv. Loss to follow up was 33% for the Iv group. |
| O’Reilly et al [27], 2016, Australia, Hybrid | A one-year Mothers After Gestational Diabetes in Australia Diabetes Prevention Program (MAGDA-DPP) in women with prior GDM within their first postnatal year versus standard care. | Individual session delivered by specially trained HCPs and included DPP overview, lifestyle modification goals (i.e. PA and diet goal setting); group sessions delivered by specially trained HCPs in a community venue and promoted understanding diabetes and risk factors, knowledge and skill building on lifestyle modification; telephone sessions comprised of a review of progress and long-term goal setting; a MAGDA-DPP handbook was provided during first DPP session. | Fidelity measures incorporated throughout Iv (facilitator manual, detailed training program, audio recording of all sessions); | One individual session; five group sessions during intensive phase; two additional follow-up telephone calls for each woman during maintenance phase. | 66% of women completed at least the individual session; 53% met program minimum exposure definition of completing individual session (n=149) and 34% had no exposure to the Iv (n=96); 13% completed only the individual session (n=37) and only 10% completed the individual session and all five group sessions (n=28). | Not reported | Retention rates were 73% for the Iv group; when pregnancy was removed from loss to follow up data, retention rates were 85% for Iv group. |
| Peacock et al [44], 2015, Australia, Hybrid | A three-month pedometer program combined with nutrition coaching, The Walking for Exercise and Nutrition to Prevent Diabetes for You (WENDY) intervention for women with history of GDM versus standard care. | Women received a pedometer linked to web-based program; encouraged to log on weekly to receive updated goals, feedback on PA and tips on diet and exercise; nutrition coaching workshop delivered by accredited dietitian; resources provided included tools to encourage portion control. | Not reported | One pedometer linked to a tailored web-based program (Stepping Up to Health); four weekly nutrition coaching workshop (four one-hour group weekly sessions). | All women randomised to the Iv group accessed the website; the mean number of pedometer uploads was 90 (SD 31); the mean recorded steps/day were 5,916 9SD 2,878, (range 5-16,645). | Not reported | Five women in the Iv group discontinued over the course of the 3-month period due to having other commitments. |
| Pérez-Ferre et al [30], 2014, Spain, In-person | A three-year Mediterranean lifestyle intervention including an educational program on nutrition and a monitored PA program in women with prior GDM versus standard care. | Women received educational info on healthy eating (adhering to a Mediterranean diet), PA and the risk of DM2 after GDM. | Not reported | 2-hr group session at first visit (7-12 weeks postpartum); group and individual sessions delivered during a period of 10 weeks between 3-6 months post-delivery; moderate intensity exercise (50-60 mins four days per week); one hr reinforcement sessions added after monitored PA period. | Not reported | Not reported | Four women in the Iv group did not complete the first annual evaluation. |
| Reinhardt et al [39], 2012, Australia, Digital | A 6-month phone-based lifestyle education intervention using MI in rural-based women with GDM versus standard care. | Self-help educational booklet mailed to women including food and PA activity diaries for self-monitoring; two accredited diabetes educators skilled in MI sessions provided phone-based sessions. | Standardisation of Iv delivery was supported by use of a facilitator’s guidebook | 10 individualised telephone-based counselling sessions for five weeks, then monthly for five months; session duration ranged from 10 to 30 mins. | Not reported | Not reported | 38 (17%) women returned consent form and completed baseline assessments; the low participation rate (17%) means generalisations from the study to the target population are difficult. |
| Rollo et al [40], 2020, Australia, Digital | A 6-month eHealth intervention ‘Body Balance Beyond’ website in women with recent GDM versus standard care. | Women randomised into three arms:  1.High personalisation (access to website, individual telehealth coaching, and text message support)  2.Low personalisation (access to website only)  3.Waitlist control; women provided with individual telehealth coaching sessions with a dietitian and exercise physiologist. | Not reported | Five website content sections:  1.Managing my risk 2.My plan  3.Eating  4.PA  5.Wellbeing  Six individual telephone counselling sessions delivered via video call (20-30 min each). | Majority of women (91%) accessed the website at least once in first three months however this fell to 57% at 3-6 months; goal setting module accessed at least once per month by 85% of women in first three months and 50% of women in months 3-6; attendance rates for coaching sessions were 100% for session one and 93% for session two; attendance for session three was lower, 80% for dietitian and 60% for exercise physiologist. | Not reported | Retention at 6 months was 80% for high personalisation group and 54% for low personalisation group. |
| Shek et al [31], 2014, Hong Kong SAR, China,  In-person | A 36-month lifestyle modifications intervention including dietary and exercise advice in Chinese women who had GDM versus standard care. | Women referred to dietitian for dietary and exercise advice; women instructed to record food intake and PA for past 5 days before returning for follow up; women received counselling sessions from dietitian; women followed up twice at 3-monthly interval, then every 6 months until total of 36 months. | Not reported | Women were delivered individual counselling sessions; quantity Not reported | Not reported | Not reported | 14 women in Iv group withdrew consent or were lost to follow up during intervention. |
| Shyam et al [32], 2013, Malaysia, In-person | A six-month low GI dietary intervention including nutrition education in Asian women with previous GDM versus standard care. | Women were taught meal planning and given dietary sheet with recommended food servings; individual nutrition sessions delivered by nutritionist; women encouraged to engage in moderate PA for 30 mins five time per week; colour coded booklets given to women and electronic interactions via SMS and email established. | Not reported | Structured one-to-one sessions were delivered; individualised dietary sheet; a GI component teaching women to substitute high GI foods with low GI foods; colour coded booklets including food refs; quantity Not reported | Food records from 34 subjects in Iv group were received and used in analysis. | Not reported | 33 women in the Iv group out of 62 randomised completed the 6 months on trial. |
| Tawfik [33], 2017, Egypt,  In-person | A nine-month health education intervention including a health belief model (HBM) on knowledge, beliefs, self-practices, and gestational and postpartum weight in women with GDM versus standard care. | Physicians provided HBM-based health education and guidance to women; explained risk factors of developing T2D to women and advice on GWG; participants also reminded of importance of postpartum screening and exclusive breastfeeding. | Not reported | HBM-based health education, quantity of sessions Not reported | Not reported | Not reported | Not reported |
| Vézina-Im et al [3], 2019, Canada, In-person | A six-month implementation intentions intervention promoting fruit and vegetable (FV) intake based on the question-behaviour effect in women at risk of GDM versus standard care. | Women were invited to attend two follow up assessments at 3- and 6-months post-intervention; FV intake was self-reported at baseline; a validated semi-quantitative food frequency questionnaire and intervention questionnaire was administered by a dietitian on site assessing FV intake | Not reported | Two visits at research centre (lasting on avg. 60-75 minutes); involving baseline assessment and completing food frequency questionnaire; a printed mailing was chosen for 3 months. | 50 participants completed baseline visit at research centre (response rate: 89.3%), 44 respondents completed and returned documents at 3-month follow up and 45 completed the last visit at the research centre at the 6-month follow up. | Not reported | Response rate was good and attrition low; only five women did not complete visit at research centre at 6 months.  Attrition rate: 12.0% at 3-month follow up  Attrition rate: 10% at 6 month follow up. |
| Zilberman-Kravits et al [34], 2018, Israel, In-person | A 24-month cultural lifestyle intervention including dietary counselling and guided PA sessions with Jewish and Bedouin post-GDM women versus standard care. | Women attended individual  counselling sessions led by a nurse and group meetings led by a dietitian and sports instructor; women given advise on increasing PA levels to at least 150 min per week; women received healthy meals during each visit; women completed a self-reported food frequency questionnaire and PA questionnaire | Not reported | Three individual 45 min lifestyle counselling sessions and four 90 min group meetings focusing on health education; verbal and printed health information flyers; women received healthy meals including low-fat products. | Over 40% of the women performed between 120 and 150 min of exercise per week. | The Iv was adapted to fit a Bedouin cultural context; lifestyle counselling sessions were delivered in Arabic by Bedouin instructors and exercise recommendations were adapted to fit Bedouin culture. | 103 women agreed to participate; the overall proportion of IG participants who were lost to follow up was 4% after 1 year and 39% after 2 years. |
