# Supplementary material for: Process Evaluations of Interventions for the Prevention of Type 2 Diabetes in Women With Gestational Diabetes Mellitus: Systematic Review
Source: Interact J Med Res. 2025 Feb 6;14:e51718. doi: 10.2196/51718 (PMC11843062; doi:10.2196/51718)
Supplement: Multimedia Appendix 6 [file ijmr_v14i1e51718_app6.docx]

| **Authors, year, country, intervention type** | **Mechanisms of Impact** | | | **Contextual Factors** | |
| --- | --- | --- | --- | --- | --- |
|  | **Participant responses to and interactions with the intervention** | **Mediators** | **Unexpected pathways or consequences** | **Barriers** | **Facilitators** |
| Borgen et al [35], 2019, Norway,  Digital | A higher number of women reported health apps made them feel more engaged with their health (84.4% in Iv group and 63.5% in the c group) | Increased engagement in women’s self-health | Not reported | OGTTs can be unpleasant, haphazard and varied follow up postpartum; lack of motivation affected number of GDM women who completed OGTT; first month post-partum can be chaotic for mothers | Not reported |
| Carolan-Olah and Sayakhot [41], 2019,  Australia, Hybrid | Not reported | Not reported | The Iv group had unusually high percentage of women attending postpartum OGTT; nearly half the women in Iv remained above pre-intervention weight due to being overweight before study | Not reported | Not reported |
| Ferrara et al [25], 2011, United States, Hybrid | Women very satisfied with Iv (97%); most (92%) would recommend Iv to others; women requested the addition of a health website alongside telephone calls and more info about health risks after GDM after receiving iv. | Not reported | Not reported | Barriers to engaging in physical activity included:  Personal and child illness; returning to work; bad weather conditions. | Not reported |
| Ferrara et al [36], 2016, United States, Digital | Not reported | Not reported | Not reported | Not reported | Not reported |
| Holmes et al [24], 2018, Northern Ireland, Hybrid | Not reported | Not reported | Not reported | Time constraints; unavailability due to child care; desire not to leave their baby. | Not reported |
| Homko et al [37], 2007, United States, Digital | Not reported | Increased psychosocial self-efficacy levels. | Not reported | Slowness of refurbished computers; competition for computer use with older children and other household members. | 17 women (53%) in Iv group had computers and Internet access prior to study. |
| Hu et al [42], 2012, China, Hybrid | Not reported | Not reported | Not reported | Not reported | Not reported |
| Kim et al [38], 2012, United States, Digital | Women in Iv group were satisfied with the intervention; acknowledged their inability to put activity changes and recommended behaviours into practice. | Not reported | Not reported | Lack of internet access; 74 women believed they were not sedentary and did not feel motivated to engage in more PA. | Not reported |
| Koivusalo et al [28], 2016, Finland, In-person | Not reported | Not reported | Not reported | Not reported | The study nurses were midwives with strong experience in counselling pregnant women; women’s confidence in the study protocol may have been increased. |
| Lipscombe et al [26], 2019, Canada, Hybrid | 73% reported satisfaction with the Iv as excellent/very good, 22% as good, and 5% as fair/poor; 97% reported they would definitely (87%) or maybe (10%) recommend the Iv to others; Iv components most liked by women were knowledge resources (39%) and coaching (29%); Iv components least liked by women were completing diet and PA records (27%) and blood tests (10%). | Not reported | Not reported | Challenges in connecting with women during busy clinic visits; lack of time due to other competing studies occurring. | Not reported |
| Liu et al [46], 2018, China, Hybrid | Not reported | Not reported | Not reported | Not reported | Not reported |
| McManus et al [43], 2018, Canada, Hybrid | Not reported | Not reported | Not reported | Lack of concern for self about risk for T2D; relief that A1C score was normal; too busy to commit time to participate in an iv. | Having partner support; higher levels of family income; personal education; breastfeeding for 3 months. |
| Nicklas et al [45], 2014, United States, Digital | Not reported | Not reported | Not reported | Not reported | Not reported |
| O’Dea et al [29], 2015, Ireland, In-person | Women expressed the program must be accessible and flexible; the online web-based programmes would work well for them so they could choose convenient times to commit; the women valued one-to-one sessions with HCPS to encourage goal-setting. | Women valued the individual sessions with HCPs when setting health goals; stress, diet self-efficacy and QoL improved. | Not reported | Child care responsibilities; lack of time; programme too long to commit to; not practicing self-care. | Support from partner; motivation to change. |
| O’Reilly et al [27], 2016, Australia, Hybrid | Not reported | Not reported | Not reported | Not reported | Not reported |
| Peacock et al [44], 2015, Australia, Hybrid | Feedback on combination of pedometer and website was positive; delivery and content of nutrition workshop were well received. | Not reported | Not reported | Lack of time; no childcare; difficulties fitting lifestyle changes into family life. | The central location of the intervention (hospital) was a familiar and convenient location for women. |
| Pérez-Ferre et al [30], 2015, Spain, In-person | Not reported | Not reported | Not reported | Not reported | Not reported |
| Reinhardt et al [39], 2012, Australia, Digital | Positive feedback included feelings of increased confidence, reassurance and value during phone sessions were received. | Reassurance given to women helped them develop own goals and attain them. | Difficulty with data retrieval from women and GPs resulting in missing baseline blood tests and self-reported data at follow-up. | Subsequent pregnancies; childcare; returning to work; family obligations; cultural customs. | Not reported |
| Rollo et al [40], 2020, Australia, Digital | The website provided useful info for 95% and 92% of women at 3 and 6 months; only a third of women found the website motivating; most women agreed telehealth coaching improved confidence for improving diet and PA habits; fewer women (22% and 31%) found text messaging as positive; women in high personalisation group (82% at 3 months and 87% at 6 months) would recommend the intervention to other women. | Confidence increased due to individual telephone counselling sessions. | Not reported | Personal & work commitments; subsequent pregnancy. | Not reported |
| Shek et al [31], 2014, Hong Kong SAR, China,  In-person | Not reported | Not reported | Not reported | Lifestyle changes due to demands of motherhood. | Not reported |
| Shyam et al [32], 2013, Malaysia, In-person | Not reported | Not reported | Not reported | Not reported | Not reported |
| Tawfik [33], 2017, Egypt,  In-person | Not reported | Not reported | Not reported | Perceived barriers and perceived self-efficacy showed relatively low improvement in comparison to the knowledge and other belief components of the HBM; around 50% of women reported a negatively high perception about barriers in relation to adopting lifestyle change behaviours | Not reported |
| Vézina-Im et al [3], 2019, Canada, In-person | Not reported | Not reported | Not reported | Lack of time; forgetfulness; not having fruit and veg at home or at the office. | Not reported |
| Zilberman-Kravits et al [34], 2018, Israel, In-person | Not reported | Social relationships developed between the women due to regular contact in the Iv group. | Not reported | The Bedouin women faced family and social norms that made it difficult to participate and change habits to do with diet and PA; transportation problems; family constraints; lack of motivation. | The women were allowed to be absent from work by employers for whole working day so they could attend study visits. |
